# The Practice of Scottish Urologists in the Assessment and Management of Fracture Risk in the Ageing Male being Treated for Prostate Cancer

**DOI:** 10.1100/tsw.2007.202

**Published:** 2007-09-28

**Authors:** Wee Sing Ngu, Derek J. Byrne

**Affiliations:** Department of Urology, Ninewells Hospital, Dundee UK DD1 9SY, UK

**Keywords:** fracture risk, prostate cancer, androgen deprivation therapy, bone mineral density

## Abstract

The aim of this study was to ascertain the practice of urologists in Scotland in the assessment and prevention of fracture risk in males starting castration-type therapy for prostate cancer. A questionnaire survey was sent to all practicing consultant urologists in Scotland. A majority of urologists, 25 (64.1%), did not consider the state of their patients' bone mineral density (BMD) before commencing castration-type therapy. The rest used various methods to assess BMD, including clinical impression alone, plain bone radiographs, and dual-energy X-ray absorptiometry (DEXA). Various methods were used in the prophylaxis and treatment of osteoporosis, including avoidance of castration type therapy and the use of bisphosphonates and bicalutamide along with castration-type therapy. This study has shown that there is no consensus as to the assessment and management of fracture risk in patients with prostate cancer commencing or on established castration-type therapy. The situation needs to be addressed with some consensus guidance.

## INTRODUCTION

For over 60 years, androgen-deprivation therapy (ADT) has been the mainstay of treatment of advanced prostate cancer[1]. More recently, it is being recommended for use earlier in the disease[2]. As a consequence of this, patients will be on ADT for many years. One important side effect of ADT is increased fracture risk and decreased bone mineral density (BMD), which has been proven by numerous studies to affect the outcome of patients with prostate cancer[3,4,5]. BMD rapidly declines within the first 6–12 months of ADT[6,7]. In addition, it does not help that a majority of patients diagnosed with prostate cancer are older men who have a rate of 0.5–1% bone loss per year[8]. Low testosterone levels as well as hypovitaminosis D also occur in this population, which explains why osteopenia or osteoporosis is present in 10–25% of men with prostate cancer even before ADT[9,10]. This, coupled with the likelihood of bony metastases leading to pathological fractures, makes increased fracture risk a dangerous side effect that can cause critical damage[11,12,13,14]. Although many studies have been carried out to determine the best form of treatment for this particular side effect, not much has been done to focus on what urologists comprehend and practice when faced with prostate cancer patients. This study looks at what the practice of Scottish urologists is to the treatment and prevention or management of fracture risk in the ageing male with prostate cancer on castration-type therapy.

## METHODS

In August 2005, a single anonymous postal questionnaire survey [Appendix 1] was sent to all practicing consultant urologists in Scotland listed in The British Association of Urological Surgeons Members Handbook 2004/2006.

To encourage response, the questionnaire took up just one side of an A4 sheet, and was accompanied by a stamped return envelope. All envelopes were sent via first class post and labelled personally to each urologist. Each return envelope was also labelled with a unique identification number to allow tracking of nonresponders and responders. This was vital, as nonresponders were sent a second and third round of questionnaires consecutively with a period of 2 months in between to encourage response. All these steps were carried out, as they had been shown to increase response rates significantly towards postal questionnaires[15].

As the questionnaires were anonymous and mailed to urologists, ethical consent was not needed for this project. This includes the Data Protection Act and Caldecott guardian approval.

Once all the replies had returned, the data were compiled and analyzed using Microsoft Office Excel 2003 and a SPSS 14.0 statistical program.

## RESULTS

Fifty-six urologists were each sent a questionnaire [Appendix 1]. Out of the 45 replies, three were wrong addresses, one urologist no longer dealt with patients with prostate cancer, seven replied after the second round of questionnaires, and three replied only after the third round of questionnaires. In total, there was a response rate of 41/52 (79%).

Out of the 41 replies, two urologists did not treat patients with castration-type therapy. Therefore, they were excluded from the data analysis.

Of the 39 urologists who did treat patients with castration-type therapy, only 14 (35.9%) of them considered the state of their patients' bone mineralization before commencing therapy, while 25 (64.1%) did not.

Fig. 1 shows the various methods used to assess bone status. There is a wide variety of methods used alone and in combination.

If a patient's bone mineralization was assessed to be poor, 11 (28.2%) urologists would not change their management plans, two (5.1%) would not use castration-type therapy alone or at all, while 19 (48.7%) would consider adding a biphosphonate to the treatment. Seven did not answer the question.

In terms of treatment, Table 1 shows a wide variety of options that are used. Of interest is the fact that nearly 60% did not answer this question.

Fifteen (38.5%) urologists would warn their patients of risk of osteoporosis and possible hip fracture and vertebral collapse before starting castration-type therapy as opposed to the 24 (61.5%) that would not.

## DISCUSSION

Due to the high response rate, 41 (79%), it is likely that these results accurately reflect the views of practicing urologists in Scotland. This study has shown that a significant proportion of Scottish consultant

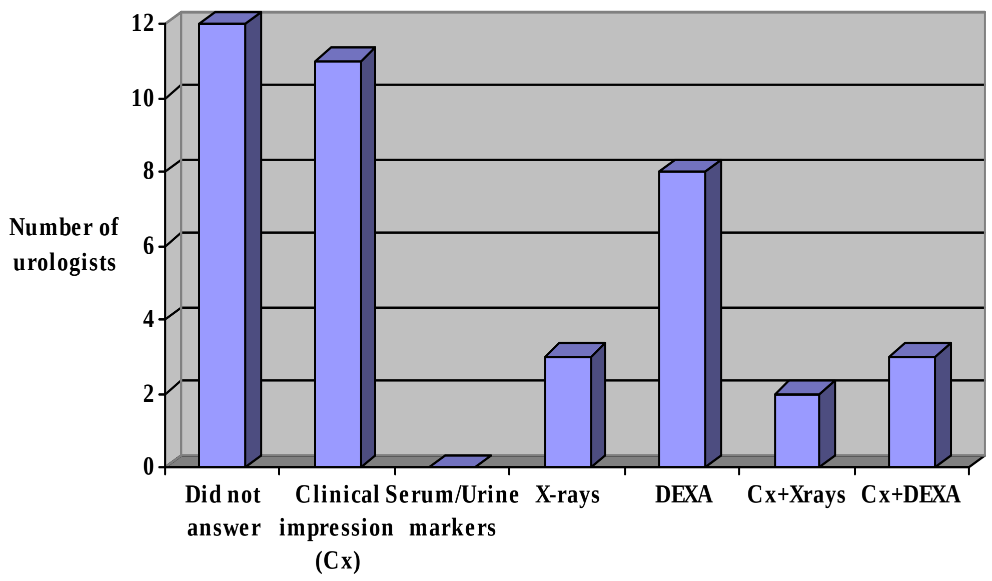

**FIGURE 1.** Methods for the assessment of bone status.

**TABLE 1**
**Alternative Therapies Used if Patient's Bone Mineral Status is Assessed to be Poor**

| | Frequency | Percentage |
|---|---|---|
| Did not answer | 23 | 59 |
| Oestrogen | 0 | 0 |
| Oestrogen and aspirin | 0 | 0 |
| Bicalutamide | 2 | 5.1 |
| Castration therapy plus bisphosphonates | 4 | 10.3 |
| Castration therapy plus vitamin D | 0 | 0 |
| Castration therapy plus calcium supplements | 2 | 5.1 |
| Others | 2 | 5.1 |
| Oestrogen and castration therapy with bisphosphonates | 1 | 2.6 |
| Oestrogen, aspirin, and bicalutamide | 1 | 2.6 |
| Bicalutamide with castration therapy plus bisphosphonates | 3 | 7.6 |
| Castration therapy plus bisphosphonates and calcium supplements | 1 | 2.6 |
| **TOTAL** | **39** | **100.0** |

urologists tend to overlook the BMD of patients before commencing ADT. Only 14 (35.9%) considered the state of a patient's bone mineralization before starting treatment. It is becoming increasingly common for men with prostate cancer to be started on hormonal therapy much earlier, as there is evidence that this would prolong survival in certain settings[16,17,18]. However, a prolonged duration of ADT would increase the risk of osteoporotic fractures and worsen the outcome of treatment. Thus, it is important that BMD is measured from the start of ADT to prevent or lessen potential complications[19].

The variety of methods used in managing patients with potential osteoporosis highlights the lack of an evidence-based consensus in this area.

This study has also shown that 15 (38.5%) urologists in Scotland would not warn patients of the risk of osteoporosis and possible hip fracture and vertebral collapse before starting castration-type therapy.

Considering the fact that many studies have shown that patients on any form of castration-type therapy, be it medical or surgical, are at a significantly increased risk of osteoporosis and fractures[12,20,21,22, 23,24,25,26,27,28], this is an important clinical governance issue. The time has come for evidence-based guidelines to be drawn up to better manage this group of patients.

## APPENDIX 1

## Questionnaire

Please circle the answer that you deem appropriate.

1. Do you treat prostate cancer patients with castration (medical or surgical) therapy? Yes No

2. Do you consider the state of these patients' bone mineralization before starting? Yes No

3. How do you assess bone mineralization status?

a. Clinical impression (e.g., diet, smoking, medication, and activity habits)
   b. Serum or urine biochemical markers
   c. Plain bone X-rays
   d. DEXA scanning

4. If you assess the patient's bone mineral status as poor, how would that alter your approach to castration (medical or surgical) therapy?

a. Would not change my management.
   b. I would not use castration-type therapy alone or at all.
   c. I would consider adding a biphosphonate to the treatment.

5. What alternative therapy would you use instead?

a. Oestrogen
   b. Oestrogen and aspirin
   c. Bicalutamide
   d. Castration therapy plus bisphosphonates
   e. Castration therapy plus vitamin D
   f. Castration therapy plus calcium supplements
   g. Other (please specify):

______________________________

______________________________

6. Before commencing castration-type therapy, do you warn patients of the risk of osteoporosis and possible hip fracture and vertebral collapse? Yes No
